# Supplementary material for: MTH1 protects platelet mitochondria from oxidative damage and regulates platelet function and thrombosis
Source: Nat Commun. 2023 Aug 10;14:4829. doi: 10.1038/s41467-023-40600-7 (PMC10415391; doi:10.1038/s41467-023-40600-7)
Supplement: Supplementary file 1 — Supplementary Information [file 41467_2023_40600_MOESM1_ESM.pdf]

Supplemental Figures

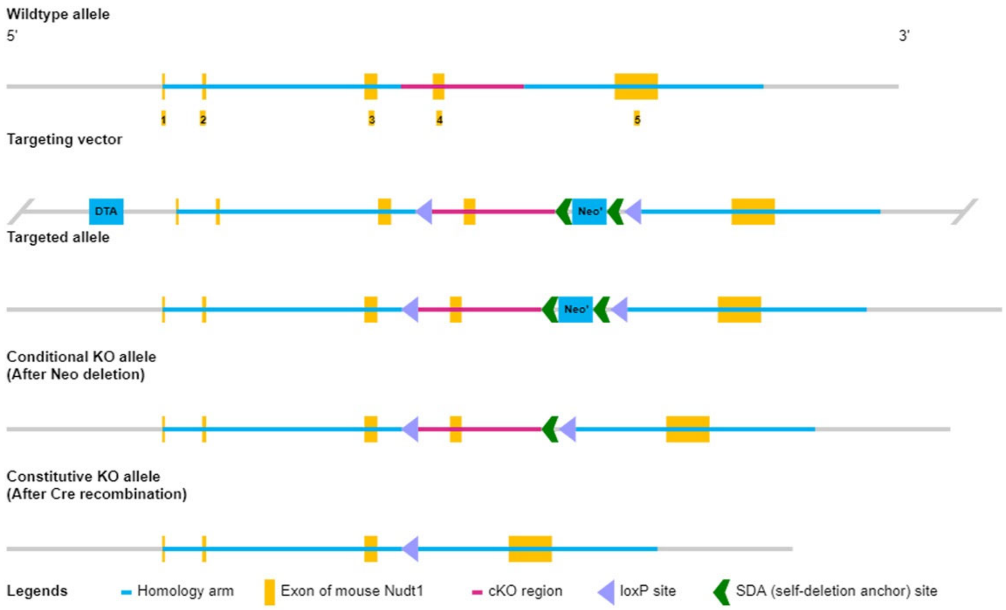

Figure S1. Schematic graph of the construction of megakaryocyte/platelet specific MTH1 knockout mice.

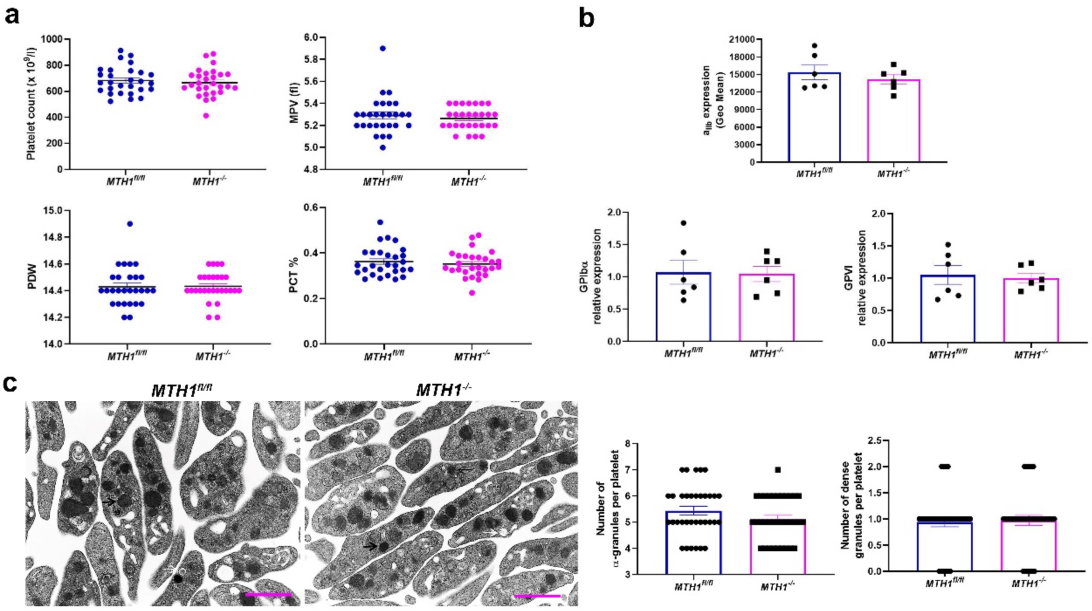

Figure S2. Platelet count, surface receptors expression and ultrastructure. (a) Platelet count, mean platelet volume (MPV), platelet distribution width (PDW), plateletcrit (PCT) detected by an automatic blood analyzer (mean± SEM, n=28 independent animals). **(b)** Surface expression of αIIbβ3 (measured by flow cytometry using FITC-conjugated anti-mouse αIIb antibody) (p = 0.4502) GPIbα (p = 0.9136) and GPVI (p = 0.7556) by quantitative real-time PCR (mean ± SEM, n = 6 independent experiments) (two-tailed unpaired student t-test). **(c)** Platelet ultrastructure (α-granules and dense granules) by electron microscopy. Scale bar: 1.0 μm (x 30,000 magnification). Black arrow: α-granule; white arrow: dense granule. The number of α-granules and dense granules was counted in 35 platelets (mean ± SEM, n=35) (two-tailed Mann-Whitney test).

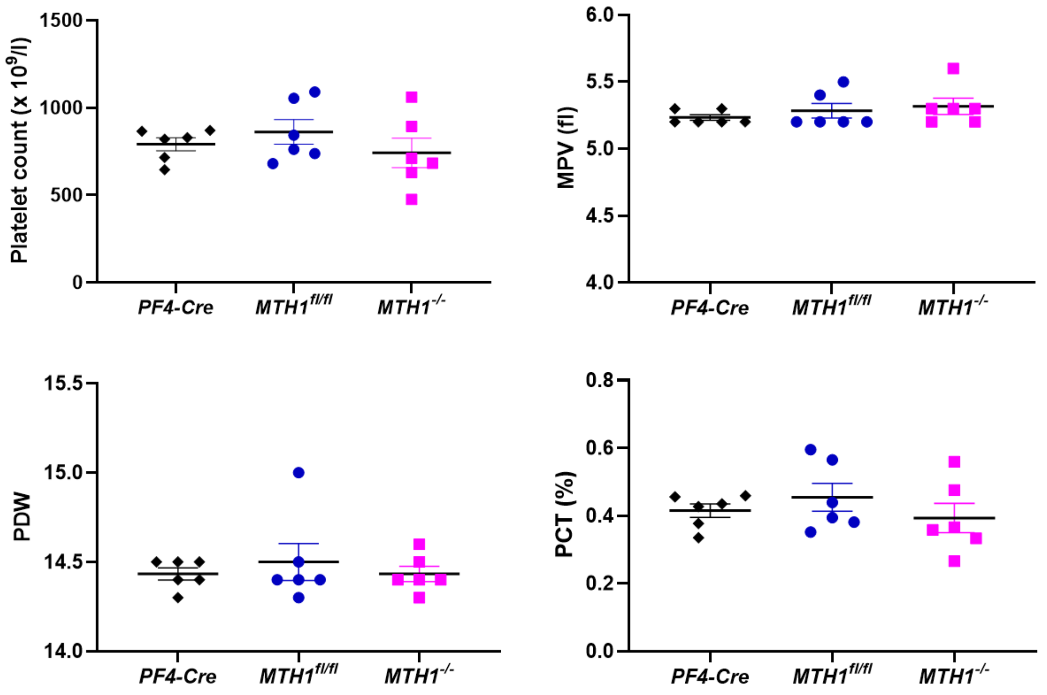

Figure S3. Platelet parameters in PF4-Cre, MTH1$^{fl/fl}$ and MTH1$^{-/-}$ mice. Blood was isolated from *PF4-Cre*, *MTH1$^{fl/fl}$* and *MTH1$^{-/-}$* mice to measure platelet count, MPV, PDW and PCT by an automatic blood analyzer (mean ± SEM, n=6 independent animals).

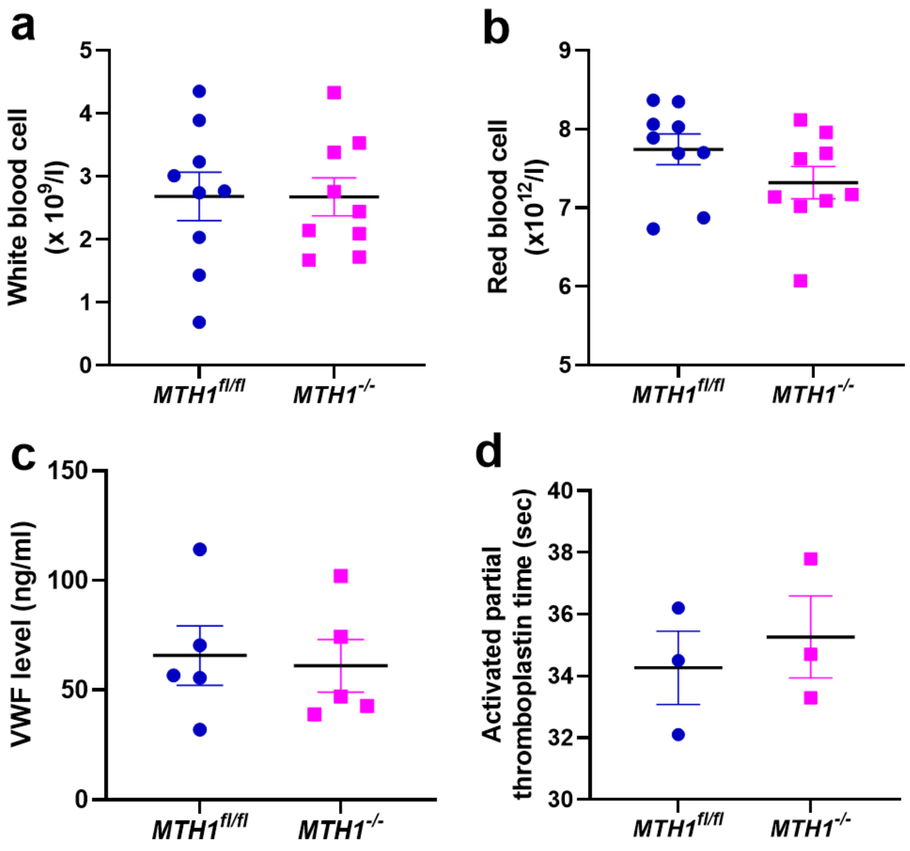

Figure S4. The number of blood cells, VWF level and coagulation activity. Blood was isolated from *MTH1*$^{fl/fl}$ or *MTH1*$^{-/-}$ mice to measure the number of white blood cell (mean ± SEM, n = 9 independent animals) **(a)** and red blood cell (mean ± SEM, n = 9 independent animals) **(b)** by an automatic blood analyzer. In addition, plasma was obtained to detect VWF level (mean ± SEM, n = 5 independent animals) **(c)** by ELISA and the activated partial thromboplastin time (mean ± SEM, n = 3 independent animals) **(d)** on an automated coagulation analyzer.

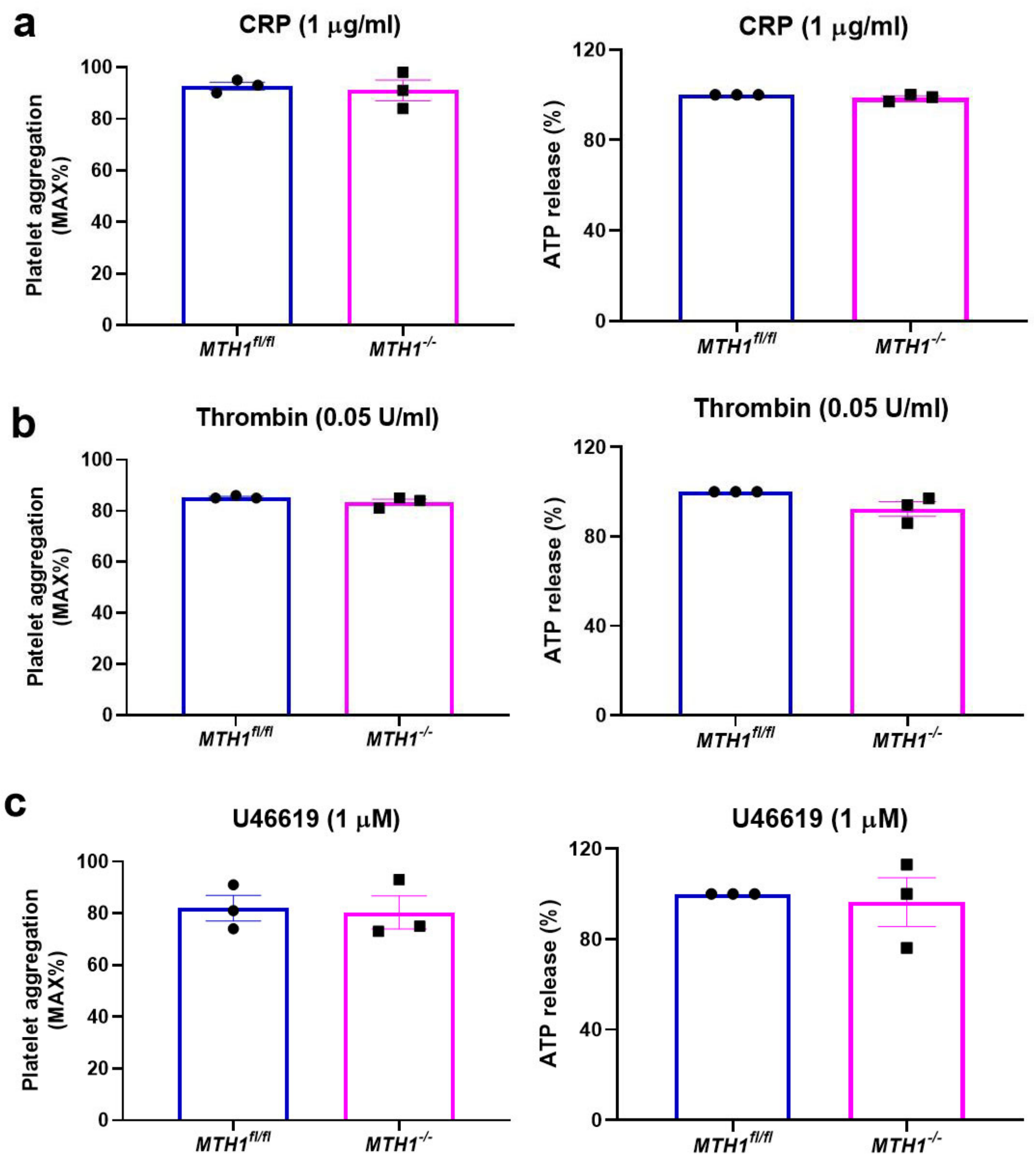

Figure S5. Platelet aggregation and ATP release. Washed platelets from *MTH1*$^{fl/fl}$ or *MTH1*$^{-/-}$ mice were stimulated with CRP (a), thrombin (b) or U46619 (c) to measure platelet aggregation and ATP release. Data were shown as mean ± SEM (n = 3 independent experiments).

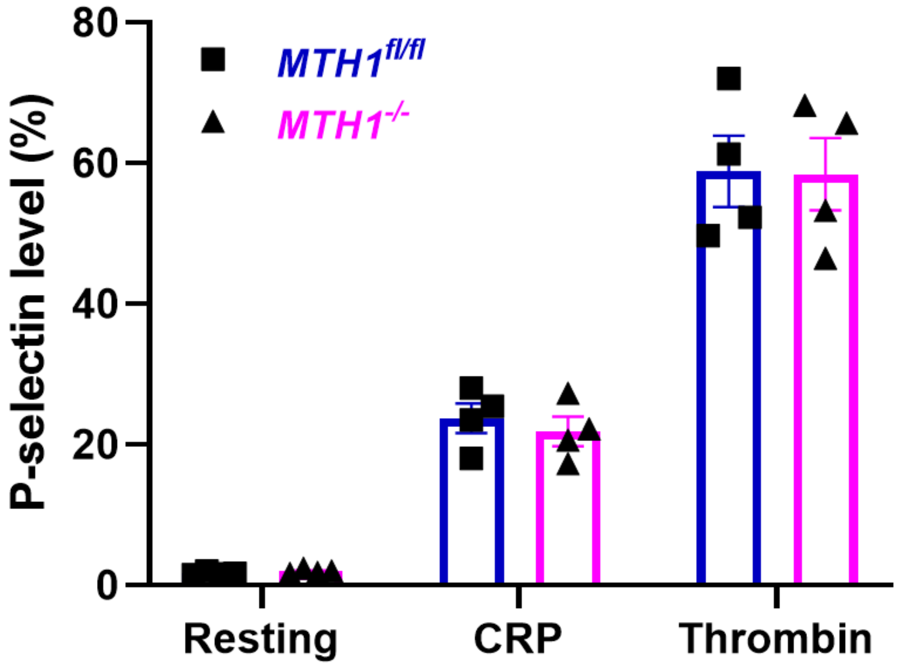

Figure S6. Platelet P-selectin expression. Washed platelets were stimulated with CRP (0.5 μg/ml) or thrombin (0.01 U/ml) followed by measuring P-selectin expression by flow cytometry. Data were shown as mean ± SEM (n = 4).

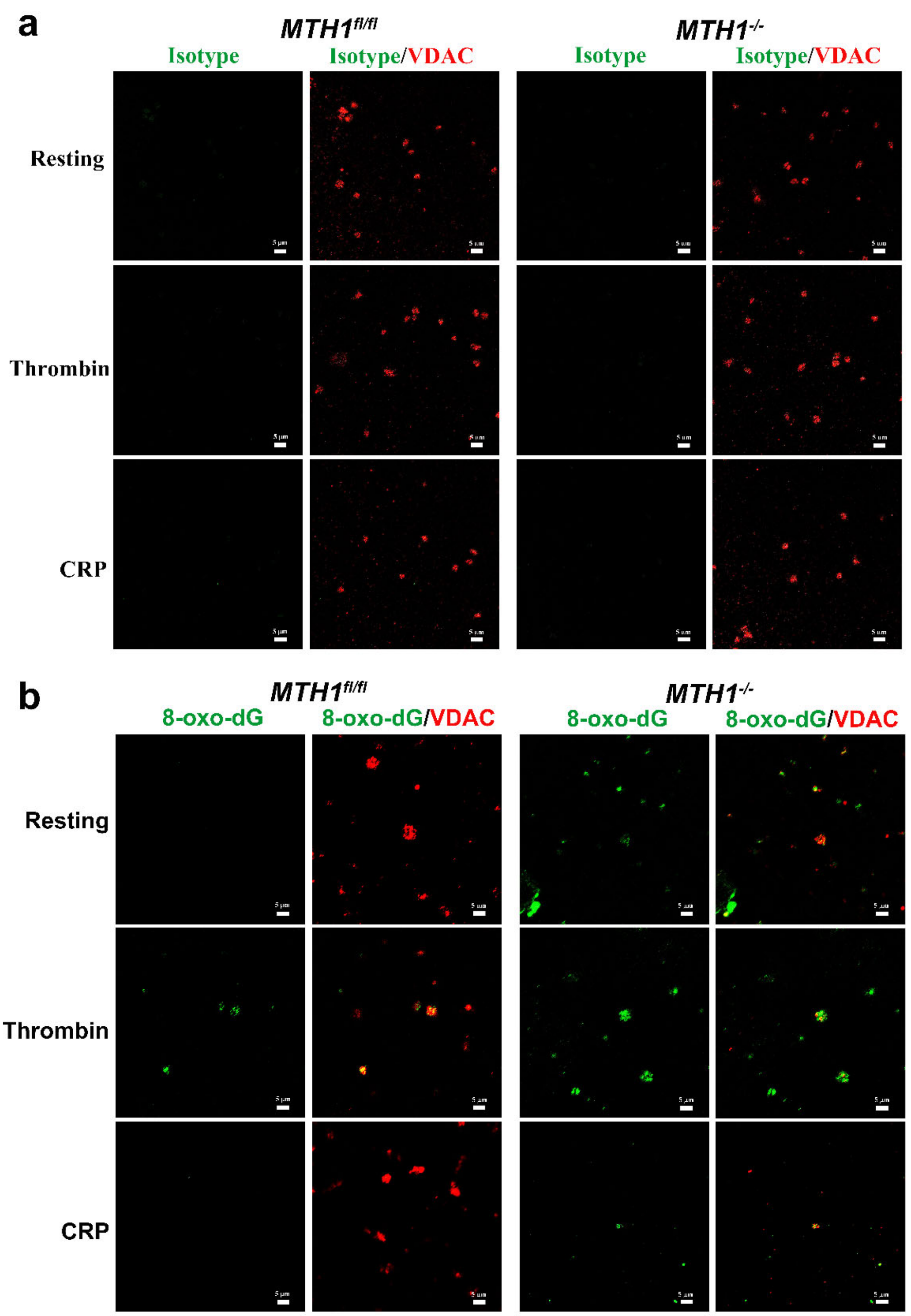

Figure S7. Accumulation of 8-OHdG in mitochondria. Mouse platelets were fixed and permeabilized followed by incubation with antibodies against isotype control antibody IgG **(a)**, VDAC or 8-OHdG **(b)** and then with subsequent fluorescence-labelled secondary antibodies. Then, platelets were mounted with Vectashield and staining was visualized under a confocal microscopy. The representative image was shown from 3 independent isolated platelets (n=3). Scale bar = 5 μm.

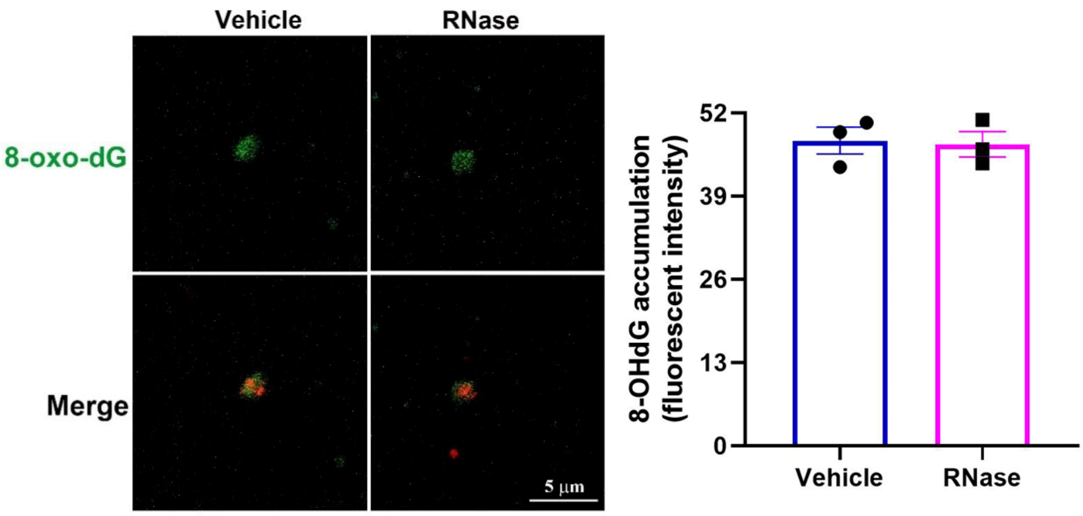

Figure S8. 8-oxo-dG staining in mouse platelets. Platelets isolated from *MTH1*$^{-/-}$ mice and stimulated with thrombin (1 U/ml) for 3 min followed by treatment with RNase A (0.02 mg/ml) for 30 minutes at 37°C. After that, platelets were stained with antibodies against with 8-oxo-dG or VDAC and the subsequent with fluorescence-labelled secondary antibodies. Then, the staining was visualized under a confocal microscopy. Representative results from three independent experiments were shown (mean ± SEM, n=3 independent experiments).

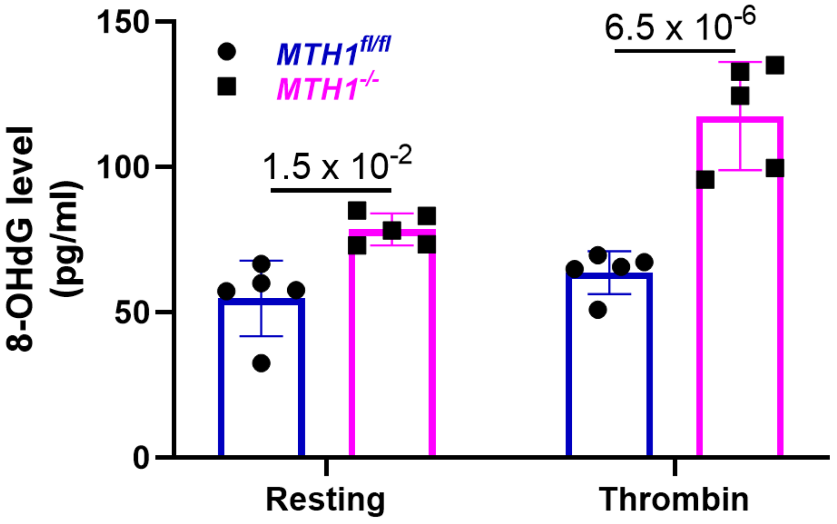

Figure S9. 8-oxo-dG level in platelets. Platelets isolated from *MTH1*$^{fl/fl}$ or *MTH1*$^{-/-}$ mice were stimulated with thrombin (1 U/ml) for 3 min to measure the level of 8-oxo-dG by ELISA. Data were shown as mean ± SD (n=5) and assessed by two-way ANOVA with Sidak multiple comparisons test.

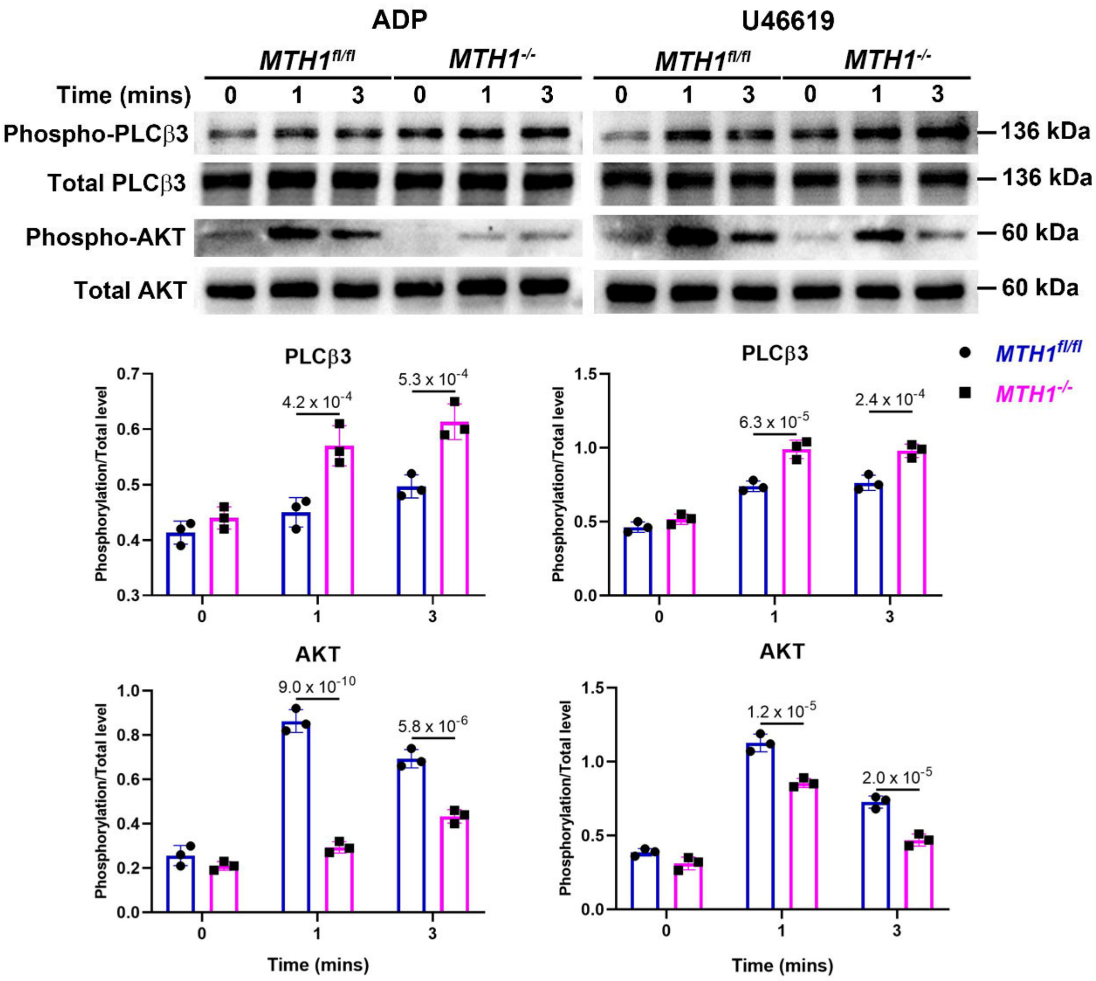

Figure S10. The phosphorylation level of PLCβ3 and AKT. *MTH1*$^{fl/fl}$ or *MTH1*$^{-/-}$ platelets were treated with ADP (10 μM) or U46619 (1 μM) for the indicated time points and then the phosphorylation level of PLCβ3 (Ser1105) and AKT (Ser473) was measured. The data were quantified based on three independent experiments (mean ± SD, n = 3, two-way ANOVA with Sidak multiple comparisons test).

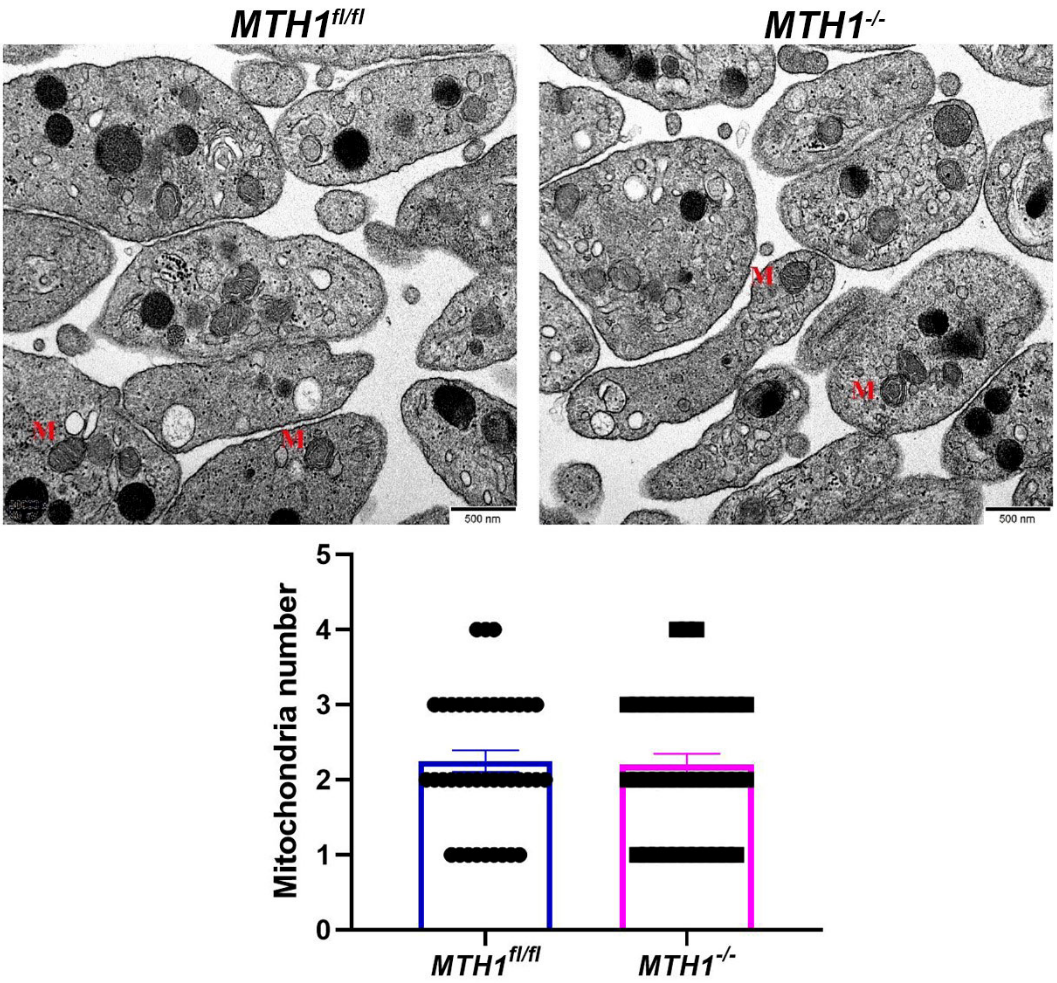

Figure S11. Electron microscopic analysis of platelet mitochondria. *MTH1*$^{fl/fl}$ or *MTH1*$^{-/-}$ platelets were fixed, dehydrated and embedded into an ultrathin section. After staining with lead citrate and uranyl acetate, mitochondria (indicated by M) was observed under a transmission electron microscope (x 30,000 magnification). Mitochondria numbers were counted in 40 platelets (mean ± SEM, n = 40, two-tailed Mann-Whitney test, p = 0.8236).

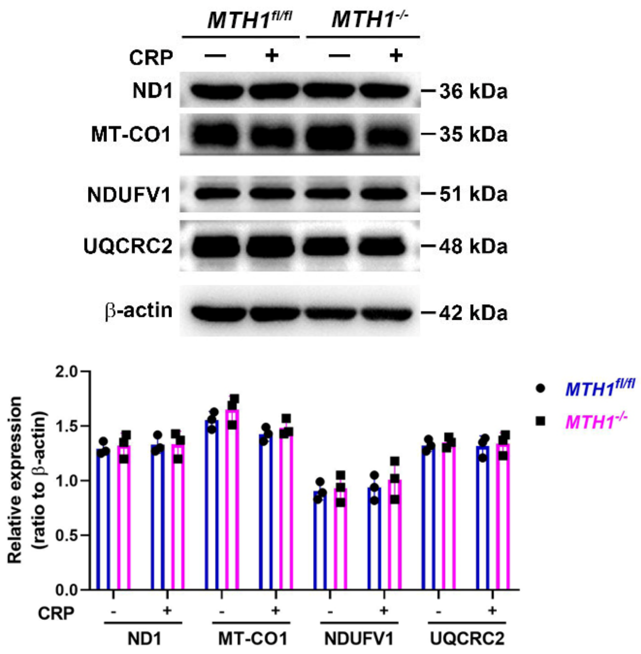

Figure S12. Expression of proteins encoded by mitochondrial DNA or nuclear DNA. Platelets were isolated from *MTH1*$^{fl/fl}$ or *MTH1*$^{-/-}$ mice and treated without (-) or with (+) CRP (5 μg/ml) for 3 mins followed by measuring the expression of ND1 (p = 0.9999; p = 0.9999), MT-CO1 (p = 0.8928; p = 0.9955), NDUFV1 (p = 0.9999; p = 0.9764), and UQCRC2 (p = 0.9999; p = 0.9999) (representative of three independent experiments) (mean ± SD, n = 3, two-way ANOVA with Sidak multiple comparisons test).

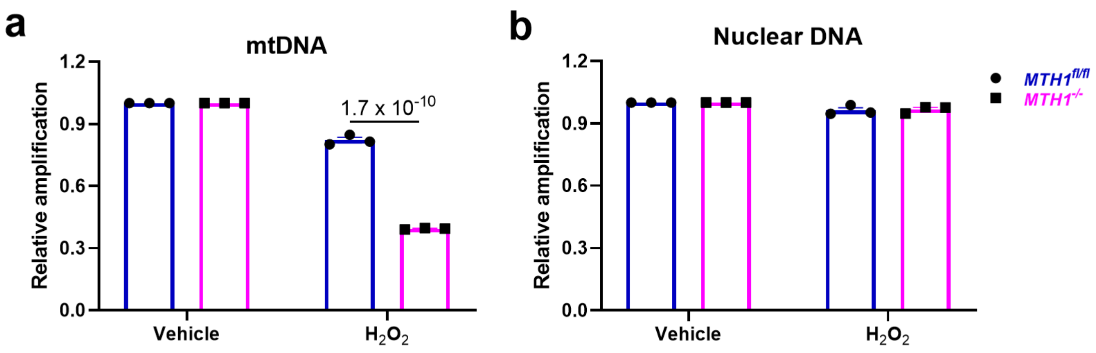

Figure S13. Assessment of oxidative damage of mitochondrial DNA and nuclear DNA. CD117^{+} hematopoietic stem cells were isolated from *MTH1*$^{fl/fl}$ or *MTH1*$^{-/-}$ mice and cultured in vitro to induce their differentiation into megakaryocytes. Then, the collected megakaryocytes were treated with H_2O_2 (50 μM) for 30 min at room temperature to induce oxidative damage followed by isolation of total DNA to measure mitochondrial DNA damage (p = 0.9999 for vehicle) **(a)** and nuclear DNA damage (p = 0.9999 for vehicle; p = 0.8906 for H_2O_2) **(b)** by quantitative PCR. The relative amplification was defined as a ratio relative to the vehicle treatment. Data were shown as mean ± SEM (n=3) and assessed by two-way ANOVA.

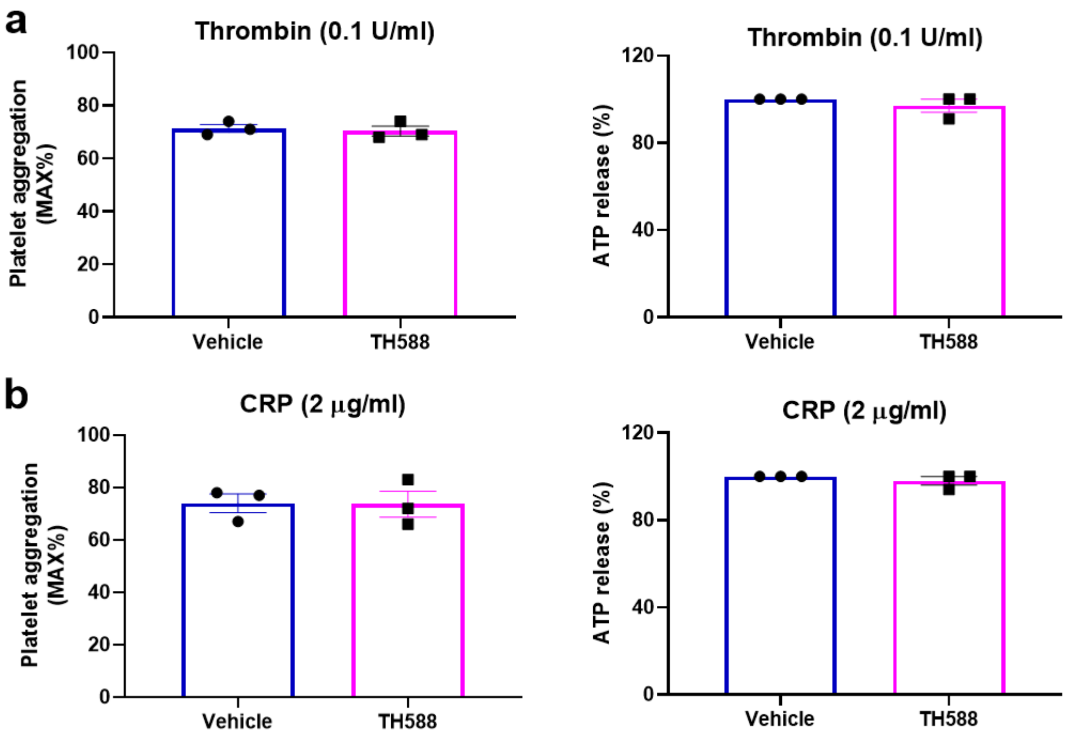

Figure S14. Platelet aggregation and ATP release in human platelets. Washed human platelets were pre-treated with 5 μM TH588 (MTH1 inhibitor) for 1 h at 37 °C followed by measuring platelet aggregation and ATP release in response to thrombin (0.1 U/ml) (mean ± SD, n = 3, two-tailed unpaired student's t-test) (p = 0.6932; p = 0.3739) **(a)** or CRP (2 μg/ml) (mean ± SD, n = 3, two-tailed unpaired student's t-test) (p = 0.9590; p = 0.3739) **(b)**.

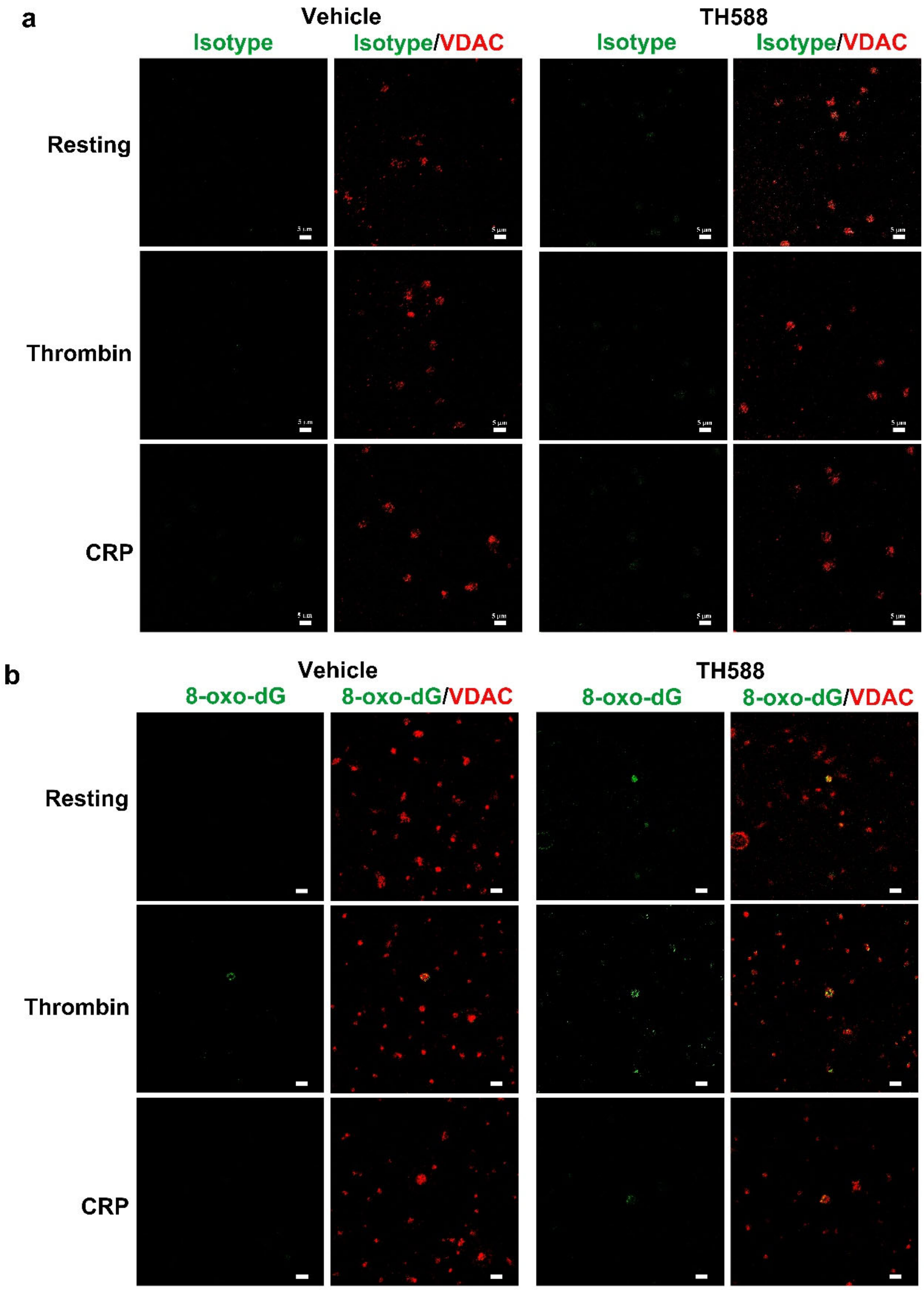

Figure S15. Accumulation of 8-OHdG in mitochondria. After TH588 treatment, human platelets were fixed, permeabilized and incubated with isotype control antibody IgG **(a)**, VDAC or 8-OHdG antibody **(b)** and then with fluorescence-labelled secondary antibodies. Platelets were mounted and staining was visualized under a confocal

microscopy. Representative images were shown from three independent experiments.

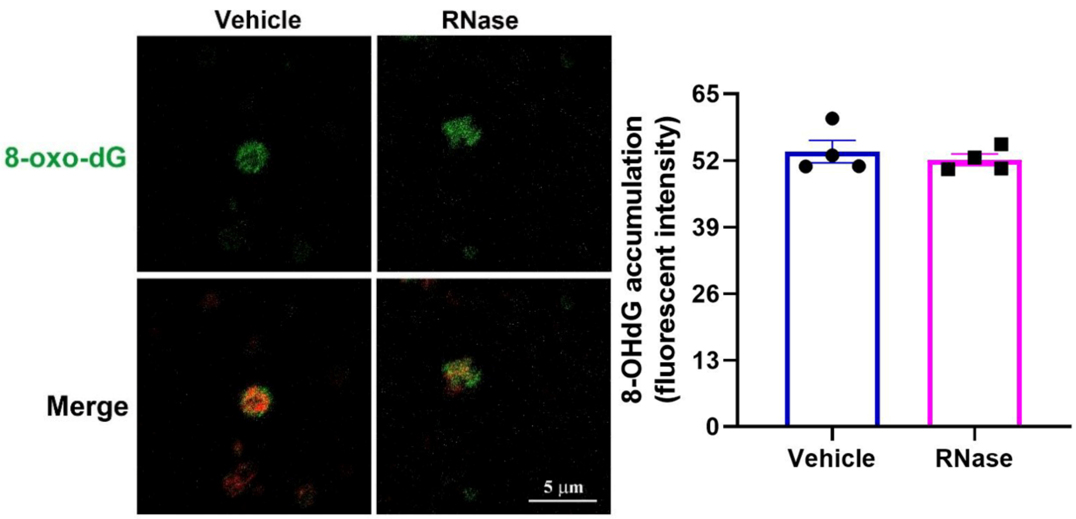

Figure S16. 8-oxo-dG staining in human platelets. Human platelets were pre-treated with 5 µM TH588 for 1 h at 37 °C and then stimulated with thrombin (1 U/ml) for 3 min followed by treatment with RNase A (0.02 mg/ml) for 30 minutes at 37°C. After that, platelets were stained with antibodies against with 8-oxo-dG or VDAC and then with secondary antibodies followed by observation of the staining under a confocal microscopy. Representative results from four independent experiments were shown (mean ± SEM, n=4, two-tailed unpaired student's t-test).

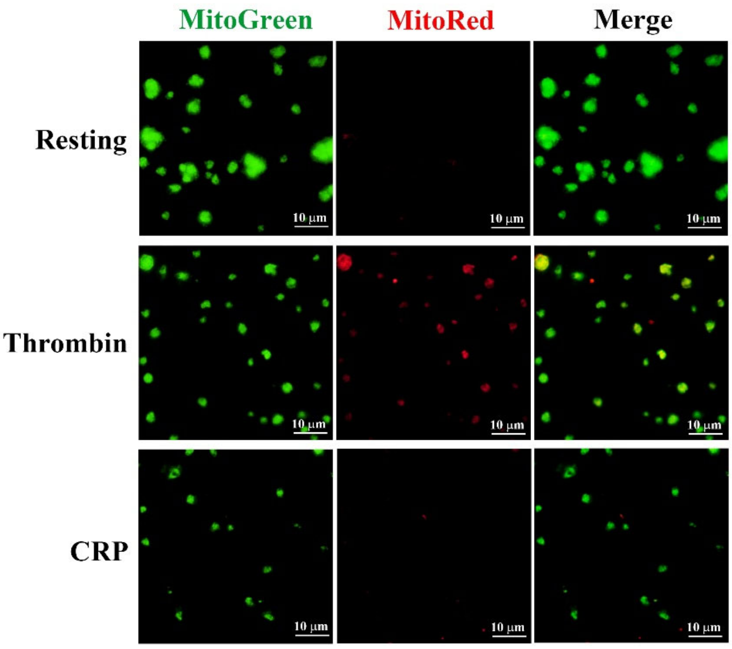

Figure S17. Mitochondrial ROS generation in human platelets. Platelets were preloaded with MitoSOX Red (5 µM) and MitoTracker Green (100 nM) for 10 min and then stimulated with thrombin (1 U/ml) or CRP (5 µg/ml) for 3 min to measured mitochondrial ROS generation by fluorescent microscope (x 100) (representative of three independent experiments).

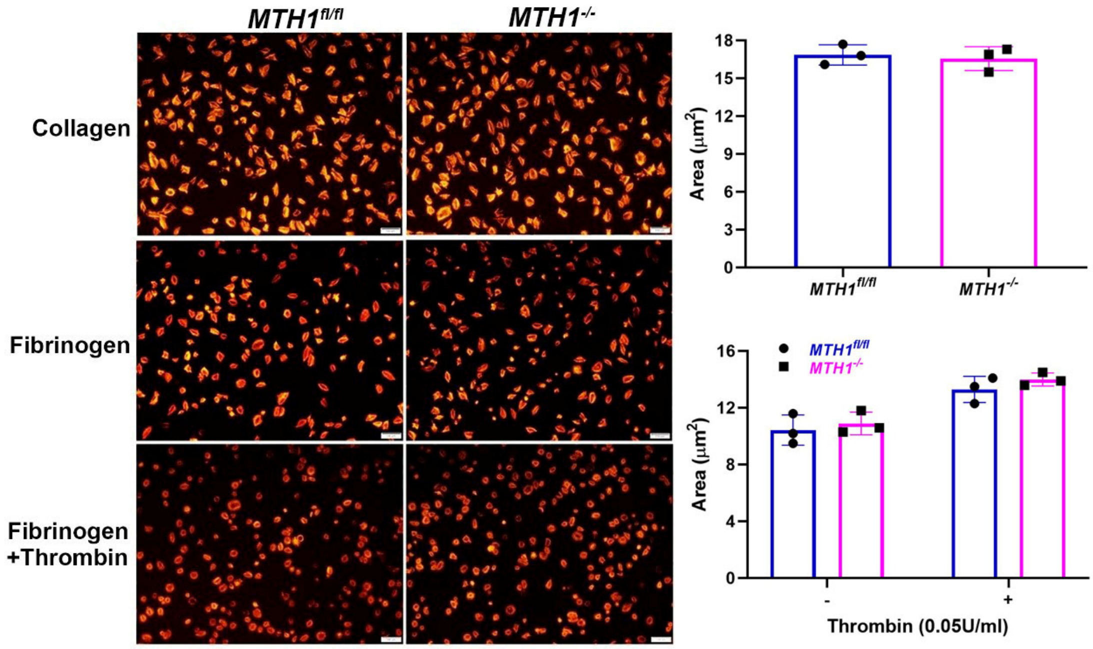

Figure S18. Platelet spreading. Platelets were placed on glass coverslips which were pre-coated with collagen or fibrinogen and then stained with Alexa Fluor-546-labelled phalloidin followed by observation under a microscope (X100) (scale bar = 20 μm) (mean ± SD, n = 3) (two-tailed unpaired student's t-test) (p = 0.6966 for collagen; p = 0.5766 for fibrinogen; p = 0.3022 for fibrinogen + thrombin).

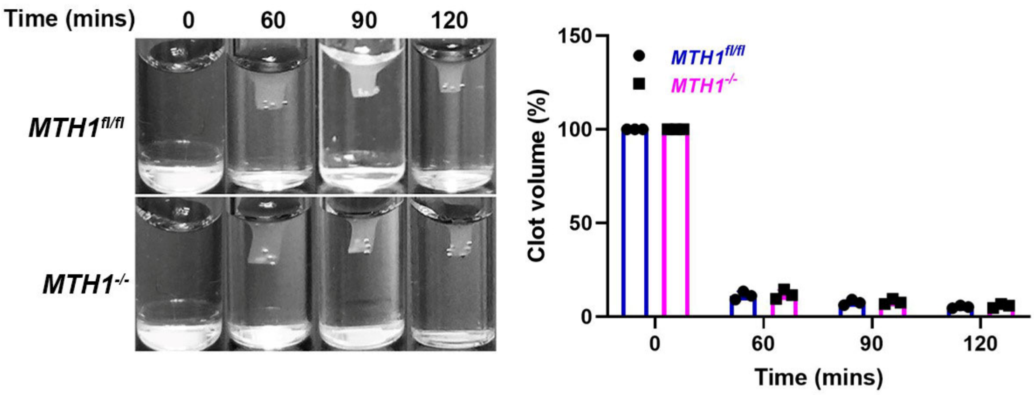

Figure S19. Clot retraction. Clot retraction was initiated after addition of thrombin (1 U/ml). Representative images at 60, 90 and 120 min. Data were quantified as clot volume (%) and presented as mean ± SD (n = 3) (Two-way ANOVA) (p = 0.9999; p = 0.9864; p = 0.9980; p = 0.9859).

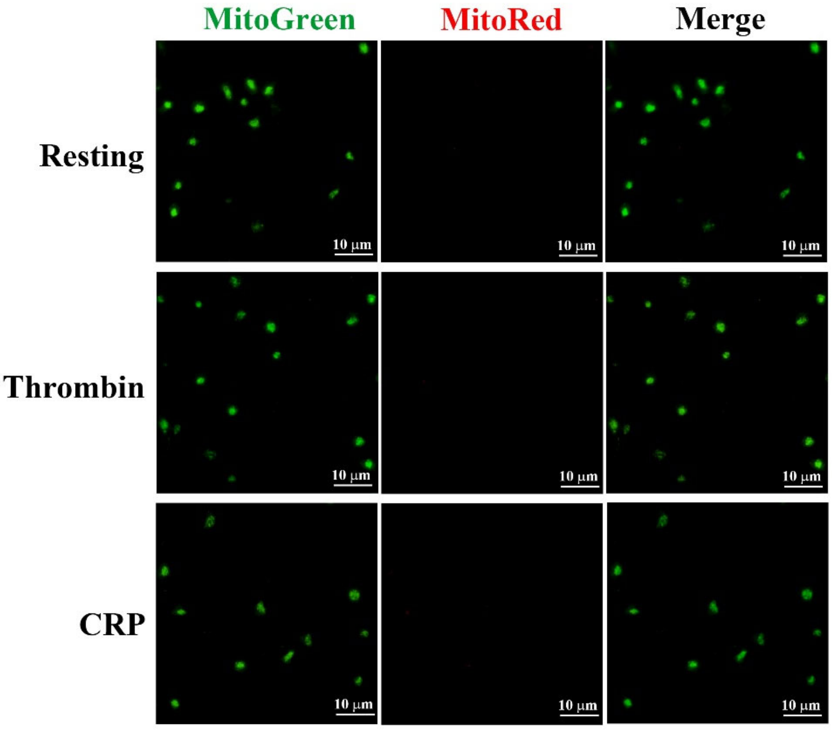

Figure S20. Mitochondrial ROS production in mouse platelets. After incubation with MitoSOX Red (5 µM) and MitoTracker Green (100 nM) for 10 min, platelets were treated with thrombin (1 U/ml) or CRP (5 µg/ml) for 1 min followed by analysis of mitochondrial ROS generation under a fluorescent microscope (x 100) (representative of three independent experiments).

Supplementary Table

Table S1. Differentially expressed phosphopeptides between control and MTH1-deficient platelets after thrombin stimulation.

Protein Names	Gene Names	Modifications in Proteins	M-B /N-B	t test p value
C5a anaphylatoxin chemotactic receptor 1	C5ar1	S328	0.513488	0.047928
Mitogen-activated protein kinase kinase kinase 3	Map3k3	S250	0.632597	0.034062
Myeloid leukemia factor 2	Mlf2	Phospho [S]	0.655886	0.019722
Store-operated calcium entry-associated regulatory factor	Saraf	S326	0.711415	0.046403
SRA stem-loop-interacting RNA-binding protein, mitochondrial	Slirp	S105	0.724269	0.003422
Pleckstrin homology domain-containing family G member 2	Plekhg2	S1277	0.72478	0.040164
Eukaryotic translation initiation factor 3 subunit D	Eif3d	S160	0.731345	0.022153
Protein EVI2B	Evi2b	S264	0.732075	0.030371
Endonuclease domain-containing 1 protein	Endod1	S498	0.732556	0.019086
Solute carrier family 22 member 23	Slc22a23	S12	0.742086	0.041613
Protein LSM14 homolog A	Lsm14a	S183	0.756182	0.011512
Ribosomal protein S6 kinase delta-1	Rps6kc1	Phospho [S]	0.78095	0.037537
Tricarboxylate transport protein, mitochondrial	Slc25a1	S157	0.796303	0.021134
Calmodulin-regulated spectrin-associated protein 1	Camsap1	S553	0.800323	0.035839
T-complex protein 1 subunit zeta	Cct6a	S246	0.808225	0.021019
Phostensin	Ppp1r18	S194	0.823012	0.048407
Phosphatidate cytidylyltransferase 2	Cds2	S59	1.203429	0.034157
Krev interaction trapped protein 1	Krit1	T151	1.224572	0.0246
Ras-related protein Rab-36	Rab36	S235	1.233705	0.015469
A-kinase anchor protein 10, mitochondrial	Akap10	S280	1.236161	0.011684
B-cell lymphoma/leukemia 10	Bcl10	S138	1.269998	0.02903
Ski oncogene	Ski	S429	1.412601	0.011619
cAMP-specific 3',5'-cyclic phosphodiesterase 4A	Pde4a	S152(100)	1.418573	0.009572

Data from 3 independent experiments were analyzed by two-tailed unpaired student's t-test.
