# Supplementary material for: MTH1 protects platelet mitochondria from oxidative damage and regulates platelet function and thrombosis
Source: Nat Commun. 2023 Aug 10;14:4829. doi: 10.1038/s41467-023-40600-7 (PMC10415391; doi:10.1038/s41467-023-40600-7)
Supplement: Supplementary file 3 — Description of Additional Supplementary Files [file 41467_2023_40600_MOESM3_ESM.pdf]

Description of Additional Supplementary Files

Supplementary Data 1: Number of phosphopeptides and phosphoproteins with phosphosites identified by the quantitative phosphorpoteimics assay.

Supplementary Data 2: Number of dysregulated phosphopeptides between two groups.
