# Supplementary material for: MTH1 protects platelet mitochondria from oxidative damage and regulates platelet function and thrombosis
Source: Nat Commun. 2023 Aug 10;14:4829. doi: 10.1038/s41467-023-40600-7 (PMC10415391; doi:10.1038/s41467-023-40600-7)
Supplement: Supplementary file 6 — Reporting Summary [file 41467_2023_40600_MOESM6_ESM.pdf]

nature portfolio

Corresponding author(s): Jianlin Qiao

Last updated by author(s): Jul 27, 2023

Reporting Summary

Nature Portfolio wishes to improve the reproducibility of the work that we publish. This form provides structure for consistency and transparency in reporting. For further information on Nature Portfolio policies, see our Editorial Policies and the Editorial Policy Checklist.

Statistics

For all statistical analyses, confirm that the following items are present in the figure legend, table legend, main text, or Methods section.

n/a	Confirmed	
☐	☒	The exact sample size (*n*) for each experimental group/condition, given as a discrete number and unit of measurement
☐	☒	A statement on whether measurements were taken from distinct samples or whether the same sample was measured repeatedly
☐	☒	The statistical test(s) used AND whether they are one- or two-sided<br>*Only common tests should be described solely by name; describe more complex techniques in the Methods section.*
☒	☐	A description of all covariates tested
☐	☒	A description of any assumptions or corrections, such as tests of normality and adjustment for multiple comparisons
☐	☒	A full description of the statistical parameters including central tendency (e.g. means) or other basic estimates (e.g. regression coefficient) AND variation (e.g. standard deviation) or associated estimates of uncertainty (e.g. confidence intervals)
☐	☒	For null hypothesis testing, the test statistic (e.g. *F*, *t*, *r*) with confidence intervals, effect sizes, degrees of freedom and *P* value noted<br>*Give P values as exact values whenever suitable.*
☒	☐	For Bayesian analysis, information on the choice of priors and Markov chain Monte Carlo settings
☒	☐	For hierarchical and complex designs, identification of the appropriate level for tests and full reporting of outcomes
☒	☐	Estimates of effect sizes (e.g. Cohen's *d*, Pearson's *r*), indicating how they were calculated

Our web collection on statistics for biologists contains articles on many of the points above.

Software and code

Policy information about availability of computer code

Data collection	No softwares are used for data collection.
Data analysis	GraphPad Prism 8.0.2, Flowjo (V10.07) and Image J (version 1.53t)

For manuscripts utilizing custom algorithms or software that are central to the research but not yet described in published literature, software must be made available to editors and reviewers. We strongly encourage code deposition in a community repository (e.g. GitHub). See the Nature Portfolio guidelines for submitting code & software for further information.

Data

Policy information about availability of data

All manuscripts must include a data availability statement. This statement should provide the following information, where applicable:

- Accession codes, unique identifiers, or web links for publicly available datasets
- A description of any restrictions on data availability
- For clinical datasets or third party data, please ensure that the statement adheres to our policy

The mass spectrometry proteomics data have been deposited to the ProteomeXchange Consortium (http://proteomecentral.proteomexchange.org) via the iProX partner repository55,56 with the dataset identifier PXD041528 (https://proteomecentral.proteomexchange.org/cgi/GetDataset?ID=PXD041528).
The authors declare that, the necessary data required to validate the findings of the paper can be found within the article itself, in the Supplementary Information or Source Data file. Source data are provided with this paper.

Human research participants

Policy information about studies involving human research participants and Sex and Gender in Research.

Reporting on sex and gender	Findings apply to male and female. Sex and gender data were not collected because the purpose of human platelet study is to evaluate the effect of the inhibition of MTH1 on platelet function through comparison of vehicle treatment with MTH1 inhibitor treatment rather than the comparison between different individuals.
Population characteristics	Human volunteers in good health, aged 20-50 years old without taking any medications during the sample collection.
Recruitment	Healthy volunteers were randomly recruited via flyer advertisements without any self-selection by the investigators. Study participants providing blood donations specifically for this research received a small financial compensation for their time, effort, and discomfort associated with the donation process.
Ethics oversight	Blood collection was conducted in accordance with the Ethnic Committee of Xuzhou Medical University. Written informed consent was obtained from all human volunteers.

Note that full information on the approval of the study protocol must also be provided in the manuscript.

Field-specific reporting

Please select the one below that is the best fit for your research. If you are not sure, read the appropriate sections before making your selection.

☒ Life sciences ☐ Behavioural & social sciences ☐ Ecological, evolutionary & environmental sciences

For a reference copy of the document with all sections, see nature.com/documents/nr-reporting-summary-flat.pdf

Life sciences study design

All studies must disclose on these points even when the disclosure is negative.

Sample size	The sample size was not predetermined by the statistical method, but based on our previous studies (eg. Wang et al., Blood 2022; Wang et al., Redox Biol 2020; Qiao et al., Haematologica 2018).
Data exclusions	No data were excluded from the analysis.
Replication	At least 3 independent replicates of all data were performed and the exact n-values are shown in the figure legends. All attempts at replication were successful.
Randomization	Samples and participants were randomly allocated into the experimental groups.
Blinding	The investigators were blinded to group allocation during data collection and/or analysis.

Reporting for specific materials, systems and methods

We require information from authors about some types of materials, experimental systems and methods used in many studies. Here, indicate whether each material, system or method listed is relevant to your study. If you are not sure if a list item applies to your research, read the appropriate section before selecting a response.

Materials & experimental systems

n/a	Involved in the study	
☐	☒	Antibodies
☒	☐	Eukaryotic cell lines
☒	☐	Palaeontology and archaeology
☐	☒	Animals and other organisms
☒	☐	Clinical data
☒	☐	Dual use research of concern

Methods

n/a	Involved in the study	
☒	☐	ChIP-seq
☐	☒	Flow cytometry
☒	☐	MRI-based neuroimaging

Antibodies

Antibodies used	All antibodies used in the flow cytometry (FC), Immunofluorescent staining or western blot (WB) are provided as follows:

Antibodies used

1. FITC Rat Anti-Mouse CD41 BD Biosciences 553847 FC:1:20
2. Anti-Human/Mouse CD62P PE Ebioscience 12-0626-82 FC:1:20
3. PE-conjugated JON/A antibody Emfret M023-2 FC:1:20
4. FITC-Annexin-V Biolegend 640906 FC:1:20
5. Anti-VDAC1 / Porin antibody Abcam ab15895 IF:1:200
6. MTH1 Antibody (H-1) Santa Cruz sc-271082 WB:1:400
7. 8-hydroxy-2'-deoxyguanosine (8-OHdG) antibody JaICA N45.1 IF:1:50
8. CD41/Integrin alpha 2b Antibody Proteintech 24552-1-AP IF:1:500
9. Alexa Fluor 488-conjugated goat anti-mouse IgG (H+L) VICMED VA1021 IF:1:100
10. Alexa Fluor 488-conjugated goat anti-rabbit IgG (H+L) VICMED VA1022 IF:1:100
11. Alexa Fluor 594-conjugated goat anti-rabbit VICMED VA027 IF:1:100
12. Alexa Fluor ® 647 AffiniPure Donkey Anti-Rat IgG (H+L) Yeasen 34413ES60 IF:1:100
13. H2DCF-DA Abcam ab113851 FC:1:200
14. MTH1 antibody Novus NB100-109SS WB:1:1000
15. MTH1 antibody Affinity Biosciences DF7359 WB:1:1000
16. Anti-MUTYH Antibody (C-6) Santa Cruz sc-374571 WB:1:400
17. ATP5A antibody (51) (CV) Santa Cruz sc-136178 WB:1:400
18. UQCRC2 antibody (G-10) (CIII) Santa Cruz sc-390378 WB:1:400
19. Anti-OGG1/2 Antibody (G-5) Santa Cruz sc-376935 WB:1:400
20. NUDT15 Polyclonal Antibody MTH2 SAB 31526 WB:1:1000
21. Rabbit Anti-NUDT18 antibody MTH3 Bioss Antibodies bs-19514R WB:1:1000
22. ND1 Polyclonal Antibody CI Proteintech 19703-1-AP WB:1:1000
23. CYTB Polyclonal Antibody (CIII) Proteintech 55090-1-AP WB:1:1000
24. ATP8 Polyclonal antibody CV Proteintech 26723-1-AP WB:1:1000
25. COX6A1 Polyclonal Antibody IV Proteintech 11460-1-AP WB:1:1000
26. NDUFV1 Proteintech 11238-1-AP WB:1:1000
27. MTCO1 Rabbit pAb (A17889)(CIV) Abclonal A17889 WB:1:1000
28. p38 MAPK (D13E1) XP® Rabbit mAb Cell Signaling Technology #8690 WB:1:1000
29. Phospho-p38 MAPK (Thr180/Tyr182) (D3F9) Cell Signaling Technology #4511 WB:1:1000
30. AKT1/2/3 Antibody Affinity AF6261 WB:1:1000
31. Phospho-Akt (Ser473) Antibody Cell Signaling Technology 9271 WB:1:1000
32. GPIbb antibody Emfret Analytics X488 in vivo platelet labeling 0.1 ug/g
33. pan-PLCbeta3 Affinity Biosciences AF4754 WB:1:1000
34. Rabbit Anti-Phospho-PLC beta3 (Ser1105) antibody Bioss Antibodies bs-3341R WB:1:1000
35. RhoA Affinity Biosciences AF6352 WB:1:1000
36. RhoA (Ser188) Affinity Biosciences AF3352 WB:1:1000
37. GAPDH Bioworld BS72410 WB:1:4000
38. B-actin Bioworld BS1002 WB:1:4000
39. Tubulin Ab-mart M30109 WB:1:4000
40. Mouse control IgG Beyotime A7028 IF: 1:100

Validation

1. FITC Rat Anti-Mouse CD41 https://www.bdbiosciences.com/zh-cn/products/reagents/flow-cytometry-reagents/research-reagents/single-color-antibodies-ruo/purified-rat-anti-mouse-cd41.553847
2. Anti-Human/Mouse CD62P PE https://www.thermofisher.cn/cn/zh/antibody/product/CD62P-P-Selectin-Antibody-clone-Psel-KO2-3-Monoclonal/12-0626-82
3. PE-conjugated JON/A antibody https://www.emfret.com/index.php?id=shop&no_cache=1&tx_feproducts_pi1%5Ba%5D=details&tx_feproducts_pi1%5BCatNo%5D=M023-2&cHash=68ee40fa8beb93387381456869f1134a
4. FITC-Annexin-V https://www.biolegend.com/en-us/products/fitc-annexin-v-5161
5. Anti-VDAC1 / Porin antibody https://www.abcam.cn/vdac1porin-antibody-mitochondrial-loading-control-ab15895.html
6. MTH1 Antibody (H-1) https://www.scbt.com/p/mth1-antibody-h-1?requestFrom=search
7. 8-hydroxy-2'-deoxyguanosine (8-OHdG) antibody https://www.jaica.com/e/products_dna_8ohdg_ab.html
8. CD41/Integrin alpha 2b Antibody https://www.ptgcn.com/products/ITGA2B-Antibody-24552-1-AP.htm
9. Alexa Fluor 488-conjugated goat anti-mouse IgG (H+L) https://www.vicmed.cn/product_details/11
10. Alexa Fluor 488-conjugated goat anti-rabbit IgG (H+L) https://www.vicmed.cn/product_details/12
11. Alexa Fluor 594-conjugated goat anti-rabbit https://www.vicmed.cn/product_details/16
12. Alexa Fluor ® 647 AffiniPure Donkey Anti-Rat IgG (H+L) https://www.yeasen.com/products/detail/1627
13. H2DCF-DA https://www.abcam.cn/dcfda--h2dcfda-cellular-ros-assay-kit-ab113851.html
14. MTH1 antibody https://www.novusbio.com/products/mth1-antibody_nb100-109
15. MTH1 antibody https://www.affbiotech.cn/goods-6153-DF7359-NUDT1_Antibody.html
16. Anti-MUTYH Antibody (C-6) https://www.scbt.com/p/mutyh-antibody-c-6?requestFrom=search
17. ATP5A antibody (51) (CV) https://www.scbt.com/p/atp5a-antibody-51?requestFrom=search
18. UQCRC2 antibody (G-10) (CIII) https://www.scbt.com/p/uqcrc2-antibody-g-10?requestFrom=search
19. Anti-OGG1/2 Antibody (G-5) https://www.scbt.com/p/ogg1-2-antibody-g-5?requestFrom=search
20. NUDT15 Polyclonal Antibody MTH2 https://www.sabbiotech.com.cn/g-202321-NUDT15-Polyclonal-Antibody-31526.html
21. Rabbit Anti-NUDT18 antibody MTH3 http://www.bioss.com.cn/prolook_03.asp?id=AF08169606023060&pro37=1
22. ND1 Polyclonal Antibody (CI) https://www.ptgcn.com/products/ND1-Antibody-19703-1-AP.htm
23. CYTB Polyclonal Antibody (CIII) https://www.ptgcn.com/products/CYTB-Antibody-55090-1-AP.htm
24. ATP8 Polyclonal antibody (CV) https://www.ptgcn.com/products/ATP8-Antibody-26723-1-AP.htm
25. COX6A1 Polyclonal Antibody (IV) https://www.ptgcn.com/products/COX6A1-Antibody-11460-1-AP.htm
26. NDUFV1 https://www.ptgcn.com/products/NDUFV1-Antibody-11238-1-AP.htm
27. MTCO1 Rabbit pAb (A17889)(CIV) https://abclonal.com.cn/catalog/A17889
28. p38 MAPK (D13E1) XP® Rabbit mAb https://www.cellsignal.cn/products/primary-antibodies/p38-mapk-d13e1-xp-rabbit-mab/8690?site-search-type=Products&N=4294956287&Ntt=%238690&fromPage=plp&_requestid=6409914
29. Phospho-p38 MAPK (Thr180/Tyr182) (D3F9) https://www.cellsignal.cn/products/primary-antibodies/phospho-p38-mapk-thr180-tyr182-d3f9-xp-rabbit-mab/4511?site-search-type=Products&N=4294956287&Ntt=4511&fromPage=plp&_requestid=6409991
30. AKT1/2/3 Antibody https://www.affbiotech.cn/goods-1869-AF6261-pan_AKT1_2_3_Antibody.html

31. Phospho-Akt (Ser473) Antibody https://www.cellsignal.cn/products/primary-antibodies/phospho-akt-ser473-antibody/9271?site-search-type=Products&N=4294956287&Ntt=9271&fromPage=plp&_requestid=6410136
32. GPIbb antibody https://www.emfret.com/index.php?id=shop&no_cache=1&tx_feproducts_pi1%5Ba%5D=details&tx_feproducts_pi1%5BCatNo%5D=X488&cHash=7c8ea4fe995c4ef8b38613f105e9aea8
33. pan-PLCbeta3 https://www.affbiotech.cn/goods-15034-AF4754-PLCbeta3_Antibody.html
34. Rabbit Anti-Phospho-PLC beta3 (Ser1105) antibody https://www.biossusa.com/products/bs-3341r
35. RhoA https://www.affbiotech.cn/goods-1905-AF6352-RhoA_Antibody.html
36. RhoA (Ser188) https://www.affbiotech.cn/goods-1513-AF3352-39. Phospho_RhoA_Ser188_Antibody.html
37. GAPDH https://bioworlde.com/Primary-Antibodies/56277.html
38. B-actin https://bioworlde.com/Primary-Antibodies/48673.html
39. Tubulin http://www.ab-mart.com.cn/page.aspx?node=%2059%20&id=%20992
40. Mouse control IgG https://www.beyotime.com/product/A7028.htm

Animals and other research organisms

Policy information about studies involving animals; ARRIVE guidelines recommended for reporting animal research, and Sex and Gender in Research

Laboratory animals	C57BL/6-Tg (Pf4-icre) Q3Rsko/J mice (Strain #: 008535) were purchased from Jackson Laboratory. C57BL/6J-Nudt1em1(flox)Cya mice (Strain #: CKOCMP-17766-Nudt1-B6J-VA) were purchased from Cyagen Biosciences Inc. C57BL/6J wide-type mice were purchased from Beijing Vital River Laboratory Animal Technology Co., Ltd. The control mice were MTH1 floxed and negative Cre recombinase with matched genetic background, age and sex. Mice of 6-10 weeks with an equal sex ratio were used in this study. All mice were housed, bred and maintained in the Laboratory Animal Center under standard husbandry conditions at Xuzhou Medical University under 12h/12h light-dark cycles, controlled temperatures (22-24 °C) and 40-50% humidity with free access to food and water. Both male and female mice were used in this study and selected using a randomized approach throughout the study. To minimize animal suffering, all possible efforts were made and mice were euthanized by CO2 inhalation. All mice were fed on a normal chow diet (#P1101F, Shanghai Pluteng Biotechnology Co., Ltd., China).
Wild animals	No wild animals were used in this study.
Reporting on sex	Sex was not considered in the study design and both male and female mice were used in this study.
Field-collected samples	The study does not involve samples collected from the field.
Ethics oversight	The experimental procedures were approved by the Animal Care and Use Committee of Xuzhou Medical University and performed in accordance the guide for the Care and Use of Laboratory Animals published by the U.S. National Institutes of Health.

Note that full information on the approval of the study protocol must also be provided in the manuscript.

Flow Cytometry

Plots

Confirm that:

☒ The axis labels state the marker and fluorochrome used (e.g. CD4-FITC).

☒ The axis scales are clearly visible. Include numbers along axes only for bottom left plot of group (a 'group' is an analysis of identical markers).

☒ All plots are contour plots with outliers or pseudocolor plots.

☒ A numerical value for number of cells or percentage (with statistics) is provided.

Methodology

Sample preparation	This study was approved by the Human Ethic Committee of Xuzhou Medical University. Informed consent has been obtained from all healthy volunteers. For preparation of human platelets, ACD-anti-coagulated venous blood was centrifuged at 120 x g for 20 min to obtain platelet-rich plasma (PRP) which was centrifuged at 1,350 x g for 15 min, washed and resuspended in Tyrode's buffer. The isolated platelets were allowed to rest for 1 hour at room temperature before use. For the recruitment of healthy volunteers, sex and gender were not collected and they were randomly recruited without any self-selection by the investigators. Mouse blood was drawn into tubes anticoagulated with trisodium citrate, glucose and citric acid (ACD) and then centrifuged to isolate platelets.
Instrument	BD LSRFortessa™
Software	FlowJo software and BD LSRFortessa™ software
Cell population abundance	More than 90% of live mouse or human platelets were validated by CD41 staining.
Gating strategy	FITC-conjugated anti-CD41a antibody was used to set the platelet gate in the Forward Scatter and Side Scatter.

☒ Tick this box to confirm that a figure exemplifying the gating strategy is provided in the Supplementary Information.
